# Malibatol A regulates microglia M1/M2 polarization in experimental stroke in a PPARγ-dependent manner

**DOI:** 10.1186/s12974-015-0270-3

**Published:** 2015-03-14

**Authors:** Jie Pan, Jia-li Jin, Hui-ming Ge, Kai-lin Yin, Xiang Chen, Li-juan Han, Yan Chen, Lai Qian, Xiao-xi Li, Yun Xu

**Affiliations:** Department of Neurology, Affiliated Drum Tower Hospital of Nanjing University Medical School, 321 Zhongshan Road, Nanjing, 210008 China; State Key Laboratory of Pharmaceutical Biotechnology, Nanjing University, 22 Hankou Road, Nanjing, Jiangsu 210093 China; Jiangsu Key Laboratory for Molecular Medicine, Nanjing University Medical School, 22 Hankou Road, Nanjing, Jiangsu 210093 China; Diagnosis and Therapy Center of Stroke in Jiangsu Province, 321 Zhongshan Road, Nanjing, Jiangsu 210008 China

**Keywords:** Experimental stroke, Inflammation, Malibatol A, Microglia, M1/M2, PPARγ

## Abstract

**Background:**

Activation of microglia plays a crucial role in immune and inflammatory processes after ischemic stroke. Microglia is reported with two opposing activated phenotypes, namely, classic phenotype (M1) and the alternative phenotype (M2). Inhibiting M1 while stimulating M2 has been suggested as a potential therapeutic approach in the treatment of stroke.

**Findings:**

In this study, we indicated that a novel natural anti-oxidant extracted from the Chinese plant *Hopea hainanensis*, malibatol A (MA), decreased the infarct size and alleviated the brain injury after mice middle cerebral artery occlusion (MCAO). MA inhibited expression inflammatory cytokines in not only MCAO mice but also lipopolysaccharide (LPS)-stimulated microglia. Moreover, treatment of MA decreased M1 markers (CD16, CD32, and CD86) and increased M2 markers (CD206, YM-1) while promoting the activation of nuclear receptor PPARγ.

**Conclusions:**

MA has anti-inflammatory effects in MCAO mice in a PPARγ-dependent manner, making it a potential candidate for stroke treatment.

## Introduction

Stroke is considered one of the leading causes of death and disability worldwide. However, to date, therapeutic options for acute ischemic stroke remain limited. Thrombolytic recombinant tissue plasminogen activator (rtPA) is the only FDA-approved drug for acute ischemic stroke that can effectively reduce the infarct size and improve functional recovery if the drug is provided within the therapeutic window of time after onset of stroke. Administration of this drug is still hard to control in a clinical setting due to the limited time window and increased incidence of intracranial hemorrhage. Consequently, a safe and effective treatment for ischemic stroke, especially at an early stage, remains challenging.

Acute inflammation plays an important role after cerebral ischemia. Brain injury provokes activation of resident microglia within minutes after ischemia, followed by infiltration of immunocytes, including neutrophils, T cells, and macrophages [1]. A plethora of pro-inflammatory mediators are triggered including TNF-α, iNOS, IL-1, and IL-6, exacerbating brain damage. The implication is that inhibiting microglia activation may represent a novel therapeutic target in acute cerebral ischemia.

In a normal brain, microglia are resident immune cells of the central nervous system which act as a sensor. Although microglia may protect the brain in some cases, the uncontrollable inflammatory responses largely overestimate its beneficial effects. It is well known that microglia, similar to periphery macrophages, could respond to micro-environmental disturbance by drastically altering phenotypes and functions. Two well-established phenotypes are the classical activation phenotype (M1) and the alternative activation phenotype (M2) [2].

Briefly, M1 microglia is a proinflammatory cellular state associated with an overexpression of inflammatory cytokines, including IL-1, TNF-α, and iNOS; whereas M2 polarized microglia state releases beneficial mediators, including IL-4, IL-10, and TGF-β, leading to homeostasis, regeneration, and neuroprotection [3]. These dual and opposing activation phenotypes, which determine the regulation of inflammatory responses after brain injury, have been found in various central nervous system diseases and injuries, including Alzheimer’s [4], multiple sclerosis [5], and traumatic brain injury [6]. Recently, they were also found to play an important role in ischemic stroke [7]. However, there is still no consensus about how microglia change phenotypes after brain injury.

The peroxisome proliterator-activated receptors (PPARs) are members of the nuclear receptor superfamily. PPARγ function relies on binding with the retinoid X receptor (RXR) to form a heterodimer complex. The complex then binds to specific DNA response elements, namely, PPAR response element (PPRE) and regulates target gene transcription [8]. PPARγ was first demonstrated as having the function of regulating adipocyte differentiation, metabolism, and glucose homeostasis [9]; for this reason, PPARγ agonist is identified as a candidate in diabetes mellitus treatment, such as troglitazone, rosiglitazone, and pioglitazone. Also, activation of PPARγ signaling has a protective role by reducing stress and inflammation in central nervous system.

Malibatol A (MA), with a molecular formula of C_28_H_20_O_7_, is a natural resveratrol oligomer extracted from the leaves of the Chinese plant *Hopea hainanensis*. It exhibits strong antioxidant effects by scavenging radicals including 2,2-diphenyl-1-picrylhydrozyl (DPPH) and O^2−^ [10]. Inoue *et al*. reported increased PPARγ expression triggered by resveratrol in mouse primary cortical cells [11]; for this reason, studies identified resveratrol as a cyclooxygenase (COX) suppressor and PPAR family activator [12]. Resveratrol has anti-inflammatory effects in the brain caused by ischemic injury [13]. According to this evidence, it is reasonable to suspect that MA, a resveratrol oligomer, has the potential to activate PPARγ in the ischemic brain. In this study, the present study aims at exploring the therapeutic effects of MA by observing the inflammatory cytokines and M1/M2 polarization markers in the cortex of the mouse experimental stroke model and also unravel the potential mechanism with PPARγ activation and the underlying mechanism.

## Materials and methods

### Middle cerebral artery occlusion model in mice

All animal experiments were approved by the Animal Care and Use Committee at Nanjing University, in Nanjing, China. Kunming mice (4 to 6 weeks,25 to 30 g, male) were provided by the Experimental Animal Center of the Drum Tower Hospital. Sixty-minute transient middle cerebral artery occlusion (MCAO) was induced by intraluminal filament technique as previously described [14]. Body temperature was maintained at 37°C ± 0.5°C with a heating lamp throughout the surgery and occlusion period. Mice were included if laser Doppler reading is below 30% of baseline and no hemorrhage were found when taking out the brain. Sham-operated mice were treated in the same way as MCAO mice, except without MCAO filament inducement.

### Treatment with MA and T0070907

MA is extracted and provided by the State Key Laboratory of Pharmaceutical Biotechnology, Nanjing University, People’s Republic of China [10]. MA was dissolved at a dose of 8 mg/mL in PBS. The mice were randomized to receive different doses of MA (5, 10, 20, and 40 mg/kg) to screening the optimum dose. Subsequently, the mice were subjected to the optimum dose of 20 mg/kg MA or the same amount of PBS by caudalvein injection within 15 min after the onset of reperfusion randomly. PPARγinhibitor T0070907 (Selleckchem) were given at a dose of 2 mg/kg by caudalvein injection 1 h before MCAO surgery. The mice were divided into five groups: sham-veh, sham-MA, MCAO-veh, MCAO-MA, and MCAO-MA-T0070907. Drugs or vehicles were arranged and labeled by an independent researcher according to the randomization plan.

### Cell culture and treatment

Mice primary microglia cells were prepared from 1 to 2 days old mice as previously described [15]. Briefly, the cerebral cortex was gently dissociated and digested in 0.25% trypsin EDTA for 10 min at room temperature and then terminate digesting using the same amount of DMEM with 10% FBS, 100 U/mL penicillin, and 100 mg/mL strexptomycin (culture medium). The resulting cells were centrifuged at 800 rpm at 37°C for 10 min. Carefully, aspirated the supernatant and resuspended the cells in the culture medium. The cells were seeded in 75-cm^2^ flasks for 10 to 12 days before the microglia cells were separated from the mixed glia cells by shaking the flasks at 180 rpm for 2 h, at 37°C. The obtained microglia cells were seeded into 6-well plates at a density of 5 × 10^5^/cm^2^for 2 to 3 days before ready for further treatment. The purity of the microglia cells was more than 95% as determined by Iba1 staining. The microglia cells were exposed to LPS from *Escherichia coli* O55:B5 (Sigma-Aldrich, St. Louis, MO, USA) at a dose of 1 μg/mL for 2 h before treated by DMEM with or without 1 μM MA for 15 h.

### Quantification of infarct size

A 2,3,5-triphenyltetrazolium chloride (TTC) assay was performed as previously described [14]. Both sides of each slice were photographed with a digital camera. The infarction size was measured by image analysis software (ImageJ, National Institutes of Health, Bethesda, MD, USA) and integrated across five slices. Mice were excluded if intracranial or subarachnoid hemorrhage was found. The infarct volume was expressed as a percentage of the contralateral side.

### Neurological severity scores

Neurological severity scores (NSS) were evaluated at 24, 48, and 72 h after MCAO as published previously [14]. Neurological function was graded on a scale of 0 to 18 (0 as normal score and 18 as maximal deficit score). NSS is a composite of motor, sensory, reflex, and balance tests. In the severity scores of injury, one point is awarded for the inability to perform a test or for the lack of a tested reflex. Therefore, a higher score means more serious injury symptoms.

### Real-time PCR

Real-time PCR was performed as described previously [16]. Briefly, the total RNA from cortex or cells was extracted using the Trizol reagent (Invitrogen, Carlsbad, CA, USA) and was reverse-transcribed into cDNA using a PrimeScript RT reagent Kit (Takara, Dalian, China) according to manufacturer’s instructions. RT-PCR was performed using quantitative PCR (ABI 7500, Thermo Fisher Scientific, Waltham, MA, USA) in the presence of a fluorescent dye (SYBR Green I; Takara Belmont, Somerset, NJ, USA). Triplicate wells were performed for each sample to obtain the cycle threshold (CT) mean, and any outlier of the triplicates was excluded if its CT value is far than 0.5 from the other two. The CT value was normalized to GAPDH of the same sample. The expression levels of mRNAs were reported as fold changes *vs*. sham-veh group. The primers (Invitrogen) were as follows:

- TNF-α: F: CAA GGG ACA AGG CTG CCC CG, R: GCA GGG GCT CTT GAC GGC AG;
- IL-1: F: AAG CCT CGT GCT GTC GGA CC, R: TGA GGC CCA AGG CCA CAG G;
- IL-6: F: GCT GGT GAC AAC CAC GGC CT, R: AGC CTC CGA CTT GTG AAG TGG T;
- iNOS: F: CAG CTG GGC TGT ACA AAC CTT, R: CAT TGG AAG TGA AGC GTT TCG;
- IL-10: F: GGT TGC CAA GCC TTA TCG GA, R: ACC TGC TCC ACT GCC TTG CT;
- TGF-β: F: GGA GCC ACA AAC CCC GCC TC, R: GCC AGC AGG TCC GAG GGG AGA; CD16: F: TTT GGA CAC CCA GAT GTT TCA G, R: GTC TTC CTT GAG CAC CTG GAT C; CD32: F: AAT CCT GCC GTT CCT ACT GAT C, R: GTG TCA CCG TGT CTT CCT TGA G; CD86: F: GAC CGT TGT GTG TGT TCT GG, R: GAT GAG CAG CAT CAC AAG GA;
- CD206: F: TTC GGT GGA CTG TGG ACG AGC A, R: ATA AGC CAC CTG CCA CTC CGG T; YM-1: F: GGG CAT ACC TTT ATC CTG AG, R: CCA CTG AAG TCA TCC ATG TC;
- GAPDH: F: GCC AAG GCT GTG GGC AAG GT, R: TCT CCA GGC GGC ACG TCA GA.

### Western blot

The procedure was used as previously published with slight modification [14]. Nuclear protein was extracted with using the Nuclear and Cytoplasmic Extract Kit (KeyGEN, Nanjing, China) according to the manufacturer’s instructions in the presence of protease inhibitor. After sodium dodecyl sulfate-PAGE electrophoresis, the samples were blotted onto polyvinylidene fluoride membrane which were then probed with related primary antibodies of PPARγ (1:500, Bioworld, Irving, TX, USA). Horseradish peroxidase-conjugated anti-rabbit secondary antibodies were then used, and the reaction was observed using chemiluminescence reagents provided with the ECL kit (Amersham Pharmacia Biotech, Piscataway, NJ, USA) and exposed to a Fuji X-ray film (Fujifilm, Tokyo, Japan). The intensity of blots was quantified by densitometry.

### Co-immunostaining

The brain slices were prepared as previously published [17]. After being blocked with 5% goat serum (TBST), the brain slices were incubated with CD16/32 antibody (1:500, BD Pharmingen, San Jose, CA, USA), CD206 (1:500, Abcam, Cambridge, UK), and Iba-1 (1:500, Wako, Richmond, VA) overnight at 4°C to 8°C. The sections were then treated with a fluroscencesecondary antibodies (1: 500, Invitrogen), and images were taken using a fluorescencemicroscope (Olympus PX51, Olympus Corporation, Shinjuku-ku, Japan) and analyzed with Adobe Photoshop 5.5 software. The cell number calculation was determined by counting of three randomly selected microscopic fields across three slides in the penumbra of ipsilateral cortex. Data are expressed as mean ± SD number of the percentage of CD16/32+ or CD206+ cells to the Iba-1+ cells.

### Electrophoretic mobility shift assay

Electrophoretic mobility shift assay (EMSA) was performed using the LightShift Chemuliminescent EMSA Kit (Thermo Scientific, Rockford, IL, USA). Briefly, equal amounts of nuclear sample were separated by 100 V on the same gel and then electrophoretically transferred to a nylon membrane at 380 mA for 30 min. The DNA was then transferred to a membrane using a UV-light cross-linking instrument (Scientz Biotechnology Co., Ltd., Ningbo City, China) with 254-nm bulbs for 45 to 60 s. Then, the biotin-labeled DNA was detected by chemiluminescence in the dark and visualized using autoradiography using Fuji X-ray film. The PPARγ probe used was F: 5'-CAA AAC TAG GTC AAA GGT CA-3', R: 3'-GTT TTG ATC CAG TTT CCA GT-5' (Invitrogen). Control EBNA system was processed at the same time.

### Chromatin immunoprecipitation

Chromatin immunoprecipitation (ChIP) assays was performed using the EZ-ChIP Kit (Millipore, Billerica, MA, USA) according to the manufacturer’s instructions. The mice brain was collected after euthanization at 24 h. Then, 37% fresh formaldehyde was added directly into the tissues at a final concentration of 1%, and they were incubated for 10 min at room temperature. The cross-linking reaction was stopped by incubation with 0.125 M glycine for 5 min. The tissues were then processed using 2 ml pre-chilled PBS containing 1× protease inhibitors. Lysis buffer was added to harvested nuclei, and chromatin was then sheared by controlled sonication. The sheared, cross-linked chromatin was then mixed with protein G agarose beads and immunoprecipitating antibody against PPARγ (1:500, cell signaling) or normal mouse IgG (as a negative control) diluted in appropriate dilution buffer overnight at 4°C. The agarose beads with bound antibody-protein-DNA complexes were washed, and ChIP-DNA fragments were purified following the manufacturer’s instructions. Finally, DNA sequences were amplified by real-time PCR using the following primers against the PPARγ site in CD206 promoter: 5'-AATCGTGGAATTTCCTCTGACA-3' (sense) and 5'-GTTTCTTCCTGGCTCTTGTCCT-3' (antisense) and YM-1 promoter: 5'-TATGCCTTTGTCCAAGTC TGA-3' (sense) and 5'-GGAAGCGAGGAAACTAGATGA-3' (antisense). PCR conditions were as described previously.

### Statistical analysis

The number of animals in each group was based on a power analysis using PASS assuming a standard deviation of 12.5% and a treatment effect of 30% (based on our own pilot study with TTC staining at 72 h) and setting *α* at 5% and 1-β at 80% (statistical power) for TTC test. The sample size thus obtained was four animals per group. The data were expressed as mean ± standard deviation (SD) and analyzed using IBMSPSS 22.0 statistical analytical. For western blot (*n* = 4), comparisons between groups were performed by Kruskal-Wallis test followed by pairwise multiple comparisons. For other experiments, comparisons between multiple groups were performed using one-way ANOVAs followed by *post hoc* Turkey HSD test. The comparative difference was considered significant at *P* < 0.05.

## Results

### Malibatol A protects against ischemic brain injury in MCAO mice

**Figure 1:**
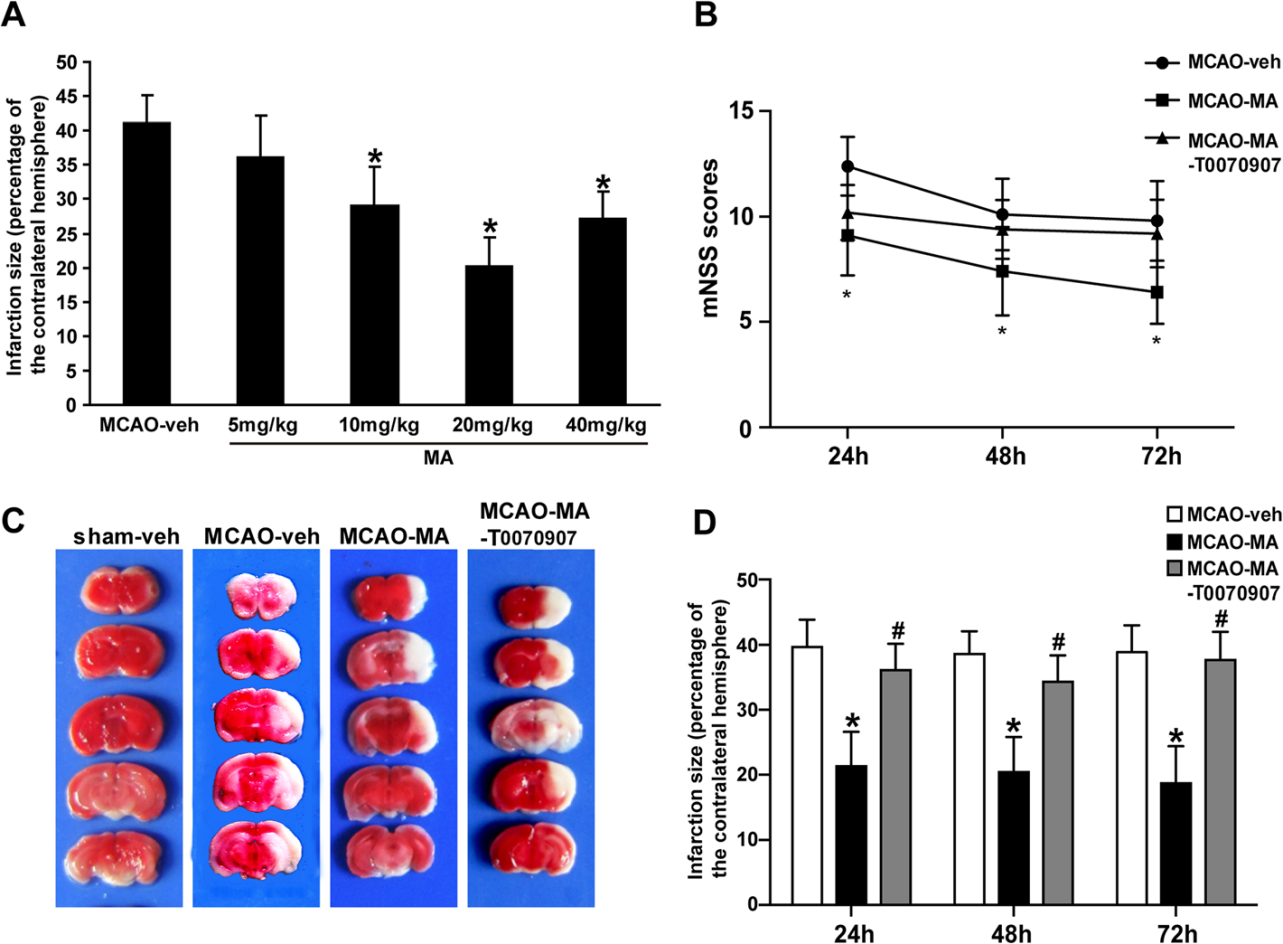
**Effects of MA on infarct volumes and neurological severity scores (NSS).** Mice were subjected to 60-min MCAO followed by treatment of vehicle (PBS) or MA. **(A)** Dose-dependent effects of MA on infarct volumes evaluated by TTC at 24 h (*n* = 6 per group).**(B)** NSSs evaluated at 24, 48, and 72 h (*n* = 10 per group).**(C)** A sample of brain slices TTC staining at 72 h after MCAO. **(D)** Effects of MA and T0070907 on infarct volumes at different time points (*n* = 10 per group). Values are mean ± SD. ^*^ *P* < 0.05 *vs*. MCAO-veh; ^#^P < 0.05 *vs*. MCAO-MA.

To evaluate the effect of MA after ischemic stroke, the infarct size of mice brains were quantified by TTC at different doses and different time points. Higher dose of MA (10 to 40 mg/kg) significantly reduced infarction (Figure 1A). The following experiments were performed at a dose of 20 mg/kg MA. Results indicated that treatment of MA significantly decreased the infarct size: 39.7% ± 4.2% in MCAO-veh *vs*. 21.3% ± 5.3% in MCAO-MA at 24 h (*P* < 0.05); 38.6% ± 3.5% in MCAO-veh *vs*. 20.4% ± 5.4% in MCAO-MA at 48 h (*P* < 0.05); 38.9% ± 4.1% in MCAO-veh *vs*.18.7% ± 5.7% in MCAO-MA at 72 h (*P* < 0.05) (*n* = 10 per group) (Figure 1D).

NSS were assessed on each mouse after ischemic injury. The change of NSS is significant between MCAO-veh and MCAO-MA treated groups: 12.4 ± 1.4 in MCAO-veh *vs*. 9.1 ± 1.9 in MCAO-MA at 24 h (*P* < 0.05); 10.1 ± 1.7in MCAO-veh*vs*. 7.4 ± 2.1 in MCAO-MA at 48 h (*P* < 0.05);9.8 ± 1.9 in MCAO-veh*vs*.6.4 ± 1.5 in MCAO-MA at 72 h (*P* < 0.05) (*n* = 10) (Figure 1B).

A total of 229 mice underwent the MCAO procedure. Of these, 12 were excluded, either for unsuccessful occlusion (CBF as measured by LDF > 30% baseline, *n* = 7) or for hemorrhagic transformation (*n* = 5), and 29 died either during (*n* = 10) or after surgery (*n* = 19). The animals that died post-surgery were evenly distributed between groups (MCAO-veh: *n* = 9, MCAO-MA: *n* = 5, MCAO-MA-T0070907: *n* = 6).

### MA-inhibited inflammatory responses in MCAO mice cortex

**Figure 2:**
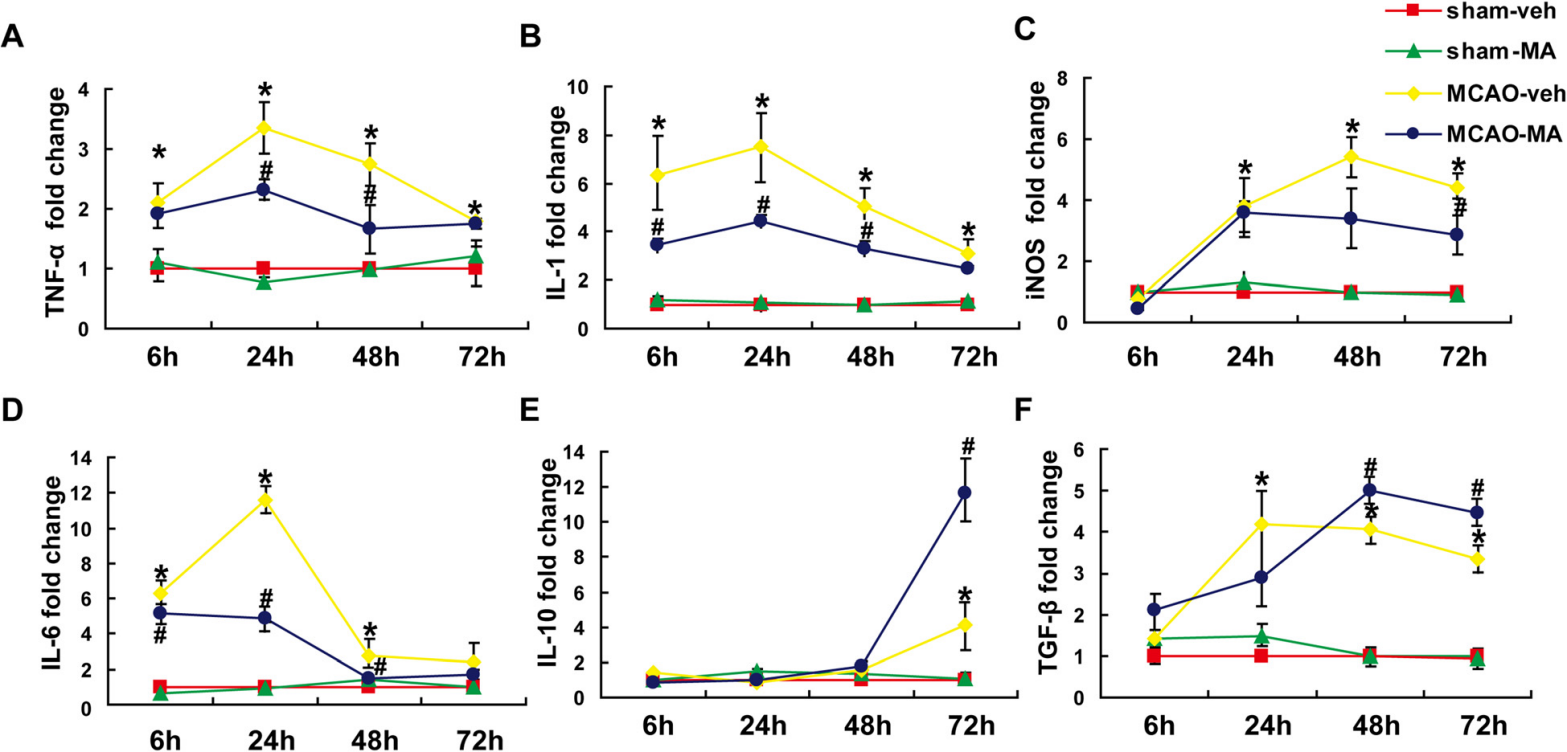
**Effects of MA on inflammatory cytokines after MCAO.** Q-PCR for mRNA expression of TNF-α **(A)**, IL-1 **(B)**, iNOS**(C)**, IL-6 **(D)**, IL-10 **(E)**, and TGF-β **(F)** on ipsilateral cortex at 6, 24, 48, and 72 h after MCAO were shown. Values are mean ± SD. ^*^ *P* < 0.05 *vs*. sham; ^#^ *P* < 0.05 *vs*. MCAO-veh;*n* = 6 in each group.

Secretion of inflammatory cytokines is a key feature after stroke. In this study, the mRNA expressions of inflammatory cytokines TNF-α, IL-1β, iNOS, and IL-6 in ipsilateral brain cortex were detected at different time points after stroke (Figure 2A,B,C,D). Generally, expressions of the cytokines were increased soon after stroke and peaked at 24 h (IL-1β, IL-6, TNF-α) or 48 h (iNOS) post-MCAO. However, in the presence of MA, the dramatic increase was blunted from 6 h after stroke, and the effect continued to 72 h, most significantly occurring at 24 and 48 h. As expected, no differences were observed between the sham-MA and the sham-veh groups.

IL-10 is regarded as a beneficial cytokine with reduced inflammation and immune reaction [18]. Interestingly, the expression of IL-10 drastically increased at 72 h by 3.8-fold in the MCAO-veh group compared to that in sham mice, and MA moved the increase forward 2.8-fold as compared to the MCAO-veh group (Figure 2E). TGF-β is believed to be important in regulation of the immune system by Foxp3+ Regulatory T cell and Th17 cells. In this study, higher level of TGF-β was discovered at 48 and 72 h in the MCAO-MA group compared with the MCAO-veh group (Figure 2F).

### MA-inhibited inflammatory responses in LPS-stimulated microglia

**Figure 3:**
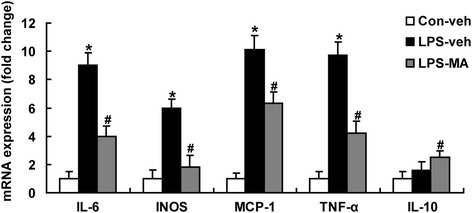
**Effects of MA on inflammatory cytokines in LPS treated microglia.** Q-PCR for mRNA expression of IL-6, iNOS, MCP-1, TNF-α, and IL-10 were shown. Values are mean ± SD. ^*^ *P* < 0.05 *vs*. con; ^#^ *P* < 0.05 *vs*. LPS; *n* = 6 in each group.

The mRNA expression of IL-6, iNOS, MCP-1, and TNF-α were increased significantly after stimulated with LPS in primary microglia and reduced markedly at 15 h after treatment of MA. The expression of IL-10 in LPS-stimulated primary microglia showed significant increase after treatment with MA compared with non-treated group (Figure 3). These *in vitro* results are in accordance with the *in vivo* ones.

### MA-promoted M2 markers and inhibited M1 markers in MCAO mice cortex

**Figure 4:**
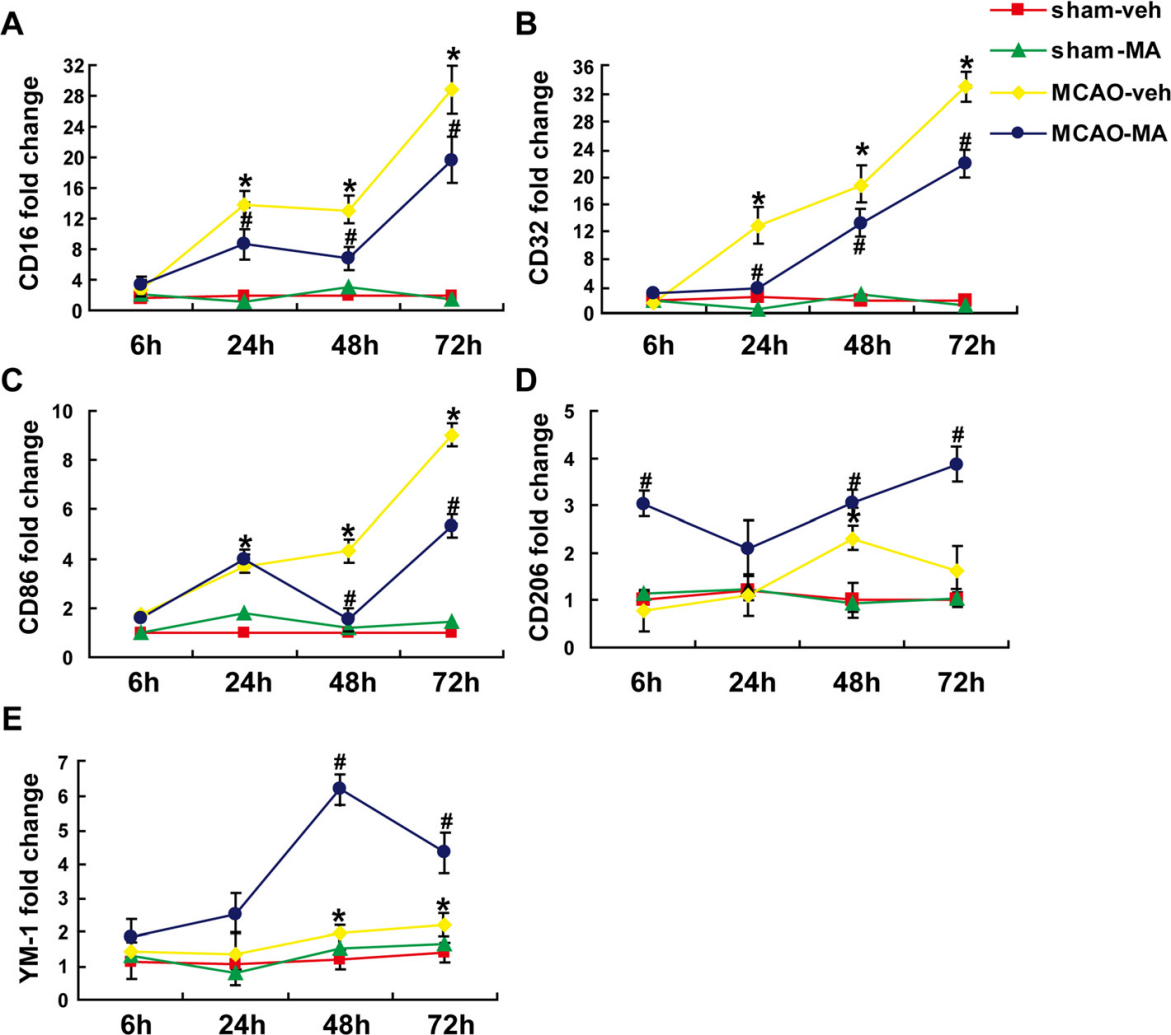
**Effects of MA on microglia/macrophage markers after MCAO.** Q-PCR for mRNA expression of CD16 **(A)**, CD32 **(B)**, CD86 **(C)**, CD206 **(D)**, and YM-1 **(E)** of ipsilateral cortex at 6, 24, 48, and 72 h after MCAO. Values are mean ± SD. ^*^ *P* < 0.05 *vs*. sham; ^#^ *P* < 0.05 *vs*. MCAO-veh;*n* = 6 in each group.

Activated microglia/macrophages are distinguished by their expression of feature genes for surface markers. Using real-time PCR, it was found that the expression of M1 markers (CD16, CD32, and CD86) and M2 markers (CD206, YM-1) were all increased after MCAO. However, the M1 markers increased much more dramatically than M2 markers. MA reversed the increasing trend of M1 markers, but stimulated M2 markers at 48 and 72 h (Figure 4A,B,C,D,E).

**Figure 5:**
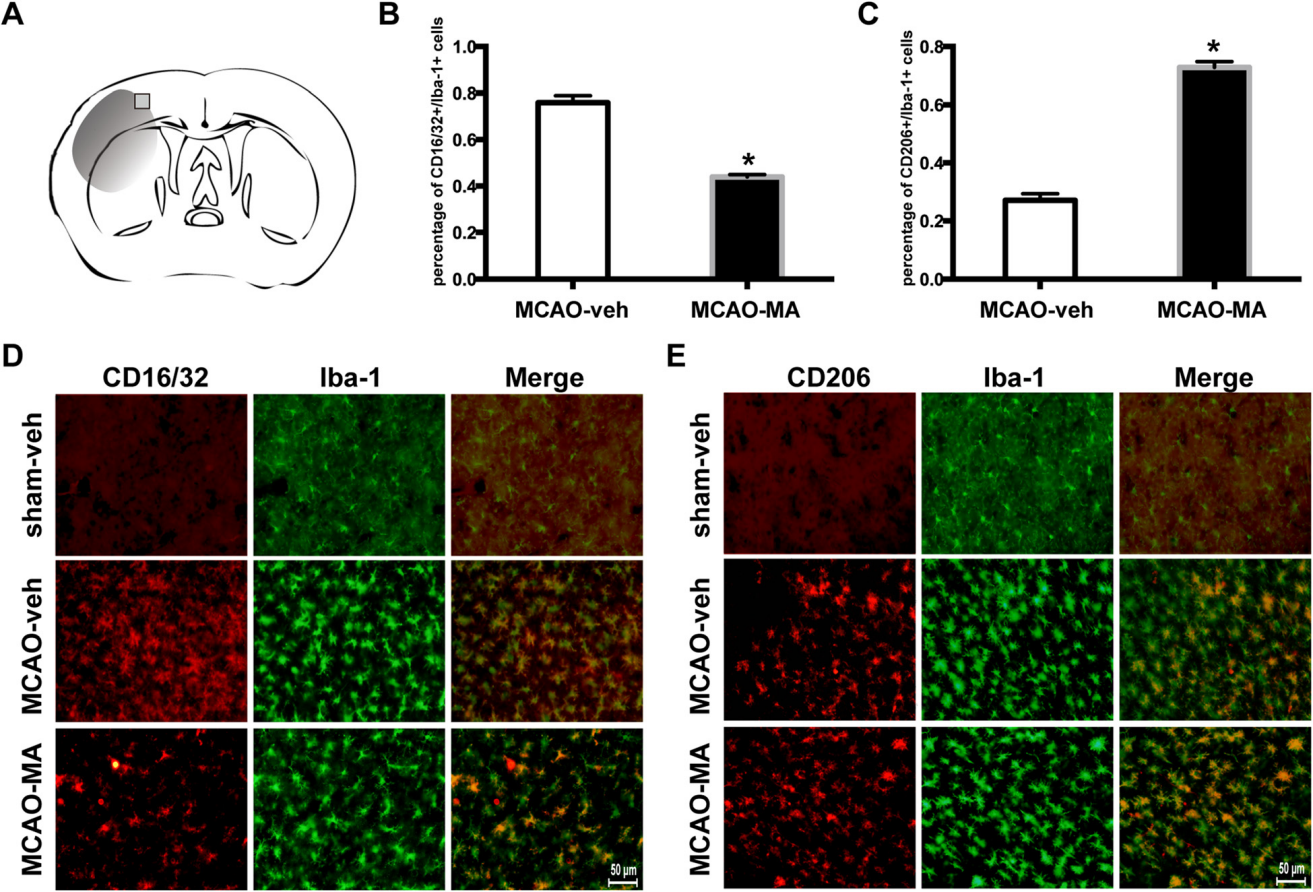
**Co-expression of Iba-1 and M1/M2 polarization markers.** Brain slices were prepared at 72 h after MCAO. **(A)** Sketch picture showing the position of the immunofluorescence pictures obtained from the penumbra of the infarct cortex. **(B)** Quantification of the percentage of CD16/32^+^/Iba-1^+^ cell. **(C)** Quantification of the percentage of CD206^+^/Iba-1^+^cell. **(D)** Cortex co-stained for CD16/32 (M1 marker) (red) and Iba-1 (green). **(E)** Cortex co-stained for CD206 (M2 marker) (red) and Iba-1 (green). Images were captured with fluorescence microscope. ^*^ *P* < 0.05 *vs*. MCAO-veh; *n* = 3 per group.

Consistent with the real-time PCR results, the expression of the M1 marker CD16/32 was highly presented in Iba1+ cells (microglia/macrophage) in MCAO mouse brain slices. The co-expression was obviously less in the presence of MA (Figure 5B,D). In contrast, the co-expression of M2 marker CD206 and Iba1 was higher in the MCAO-MA group compared with the MCAO-veh group (Figure 5C,E). The results suggest that microglia/macrophage largely activate into classical M1 phenotype without intervention. However, MA influenced the polarization process by promoting a beneficial opponent, M2 phenotype.

### MA stimulated PPARγ nuclear translocation in MCAO mice cortex

**Figure 6:**
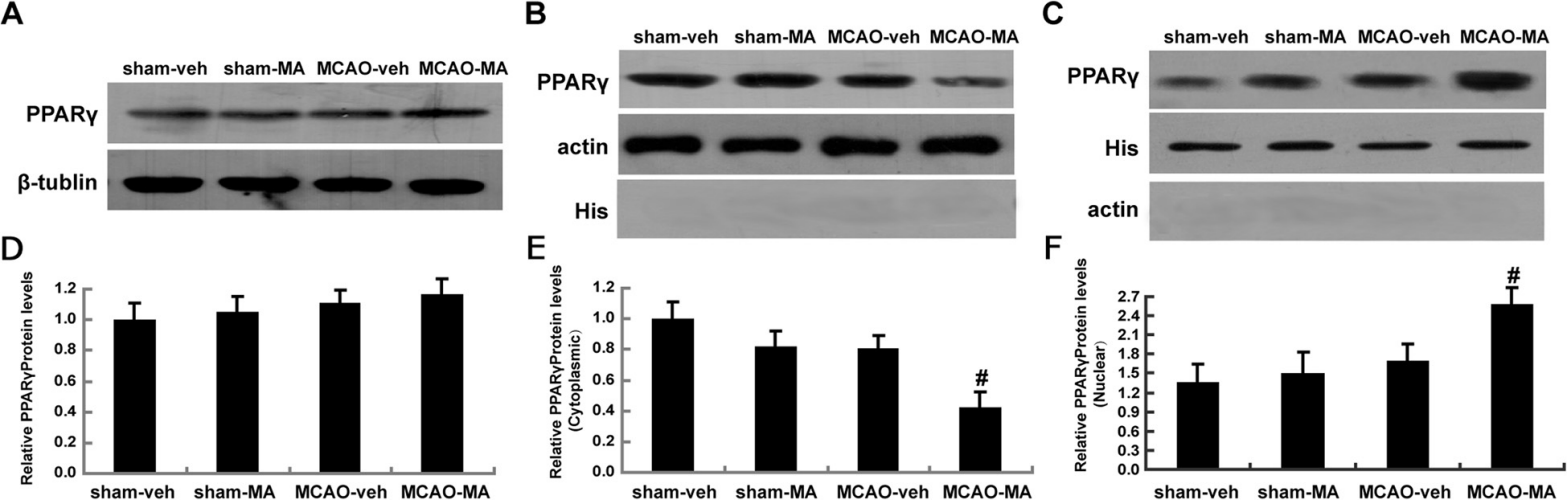
**MA stimulated PPARγ** **nuclear translocation.** Western blot of PPARγ expression in whole ipsilateral cortex tissue **(A)**, cytoplasmic **(B)**, and nuclear **(C)**. The autoradiograms are representative of three independent experiments. Quantification of PPARγ level in whole ipsilateralcortex **(D)**, cytoplasmic **(E)**, and nuclear **(F)** was expressed as the fold change compared to sham group normalized with respect to the expression of β-tubulin (whole protein),β-actin (cytoplasmic) or histone (nuclear). ^#^ *P* < 0.05 *vs*. MCAO-veh, *n* = 4 in each group.

To explore whether PPARγ plays a role in the mechanism of MA treatment, the expression of PPARγ was tested in the ipsilateral brain cortex at 24 h after MCAO using western blot. It is pointed out that PPARγ is expressed in the normal brain tissue although at a very low level [19]. In the study, expression of PPARγ was significantly reduced in cytoplasm but was increased in nuclei in the MCAO-MA group compared with the MCAO-veh group, indicating nuclear translocation of PPARγ after MA treatment (Figure 6B,C). Interestingly, the total PPARγ expression did not show much difference among different groups (Figure 6A).

### MA enhanced transcriptional activity of PPARγ in MCAO mice cortex

**Figure 7:**
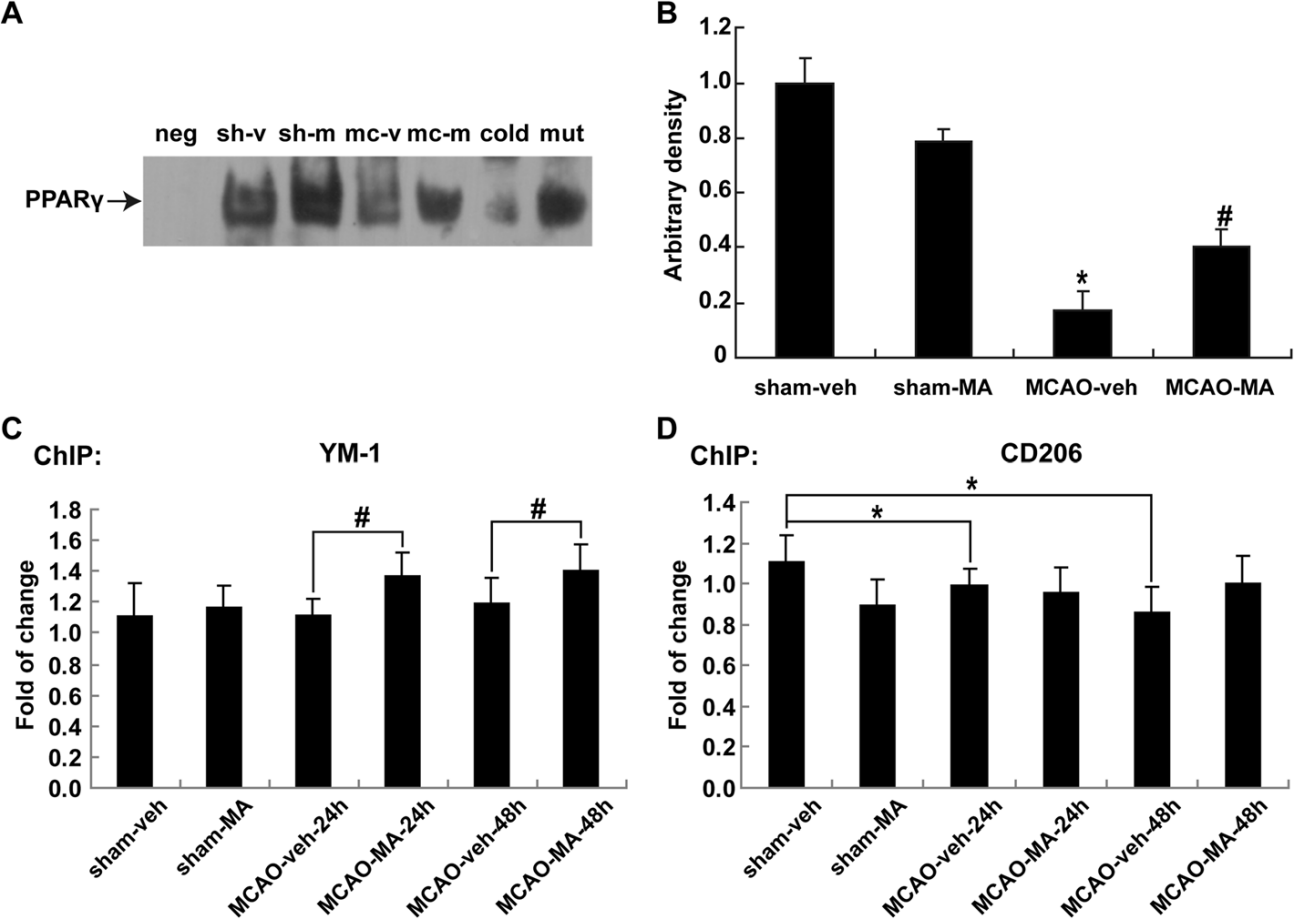
**MA increased PPARγ** **transcriptional activity.** Cortex lysates were from post-MCAO mice brains at 24 h after MCAO. **(A)** Activation of PPARγ was analyzed by EMSA at 24 h after MCAO using specific PPRE probes as described in the ‘Materials and methods’ section. The abbreviations above the lines represent the following: neg, negative control; sh-v, sham-veh; sh-m, sham-MA; mc-v, MCAO-veh; mc-m, MCAO-MA; cold, positive control + 200-fold cold probe; mut, positive control + 200-fold mutation probe. **(B)** Quantification of PPARγ level was expressed as the fold change compared to the sham group. **(C**, **D)** Binding activity of PPARγ with YM-1 **(C)** and CD206 **(D)** promoter after MCAO was detected by ChIP assay with a PPARγ antibody at 24 and 48 h. Quantifications were expressed as the fold change compared to sham group.^*^ *P* < 0.05 *vs*. sham; ^#^ *P* < 0.05 *vs*. MCAO-veh; *n* = 6 in each group.

PPARγ activation is positively related to M2 genes expression. To investigate whether MA alters M2 gene expression by accelerating PPARγ transcriptional activity, electrophoretic mobility shift assay (EMSA) was conducted at 24 h after MCAO. PPARγ-DNA binding activity was significantly decreased in MCAO group compared to sham, but was reversed in the presence of MA (*P* < 0.05) (*n* = 6) (Figure 7A).

To investigate whether M2 genes CD206 and YM-1 are the direct PPARγ target genes, ChIP assays were performed using primers from PPARγ binding sites in CD206/YM-1 promoter. The binding activity of YM-1 promoter was increased in the MCAO-MA group in post-MCAO at 24 and 48 h compared to the MCAO-veh group (Figure 7C). However, no significant changes were observed in the binding activity of CD206 in this study (Figure 7D).

### PPARγ inhibitor T0070907 switched the protective effect of MA in a M1/M2 dependent manner

Mice with PPARγT0070907 inhibitor given 1 h before MCAO surgery have significantly larger infarct size at 24, 48, and 72 h after MCAO compared with MA alone treated group. The infarct sizes of MCAO-MA-T0070907 group are as follows: 36.1% ± 4.1% at 24 h, 34.3% ± 4.1% at 48 h, and 37.7% ± 4.3% at 72 h (*P* < 0.05) (*n* = 10). The NSSs of MCAO-MA-T groups are as follows: 10.2 ± 1.3 at 24 h, 9.4 ± 1.4 at 48 h, and 9.2 ± 1.6 at 72 h (*P* < 0.05) (*n* = 10). No significant differences were demonstrated between the MCAO-veh and MCAO-MA-T groups.

**Figure 8:**
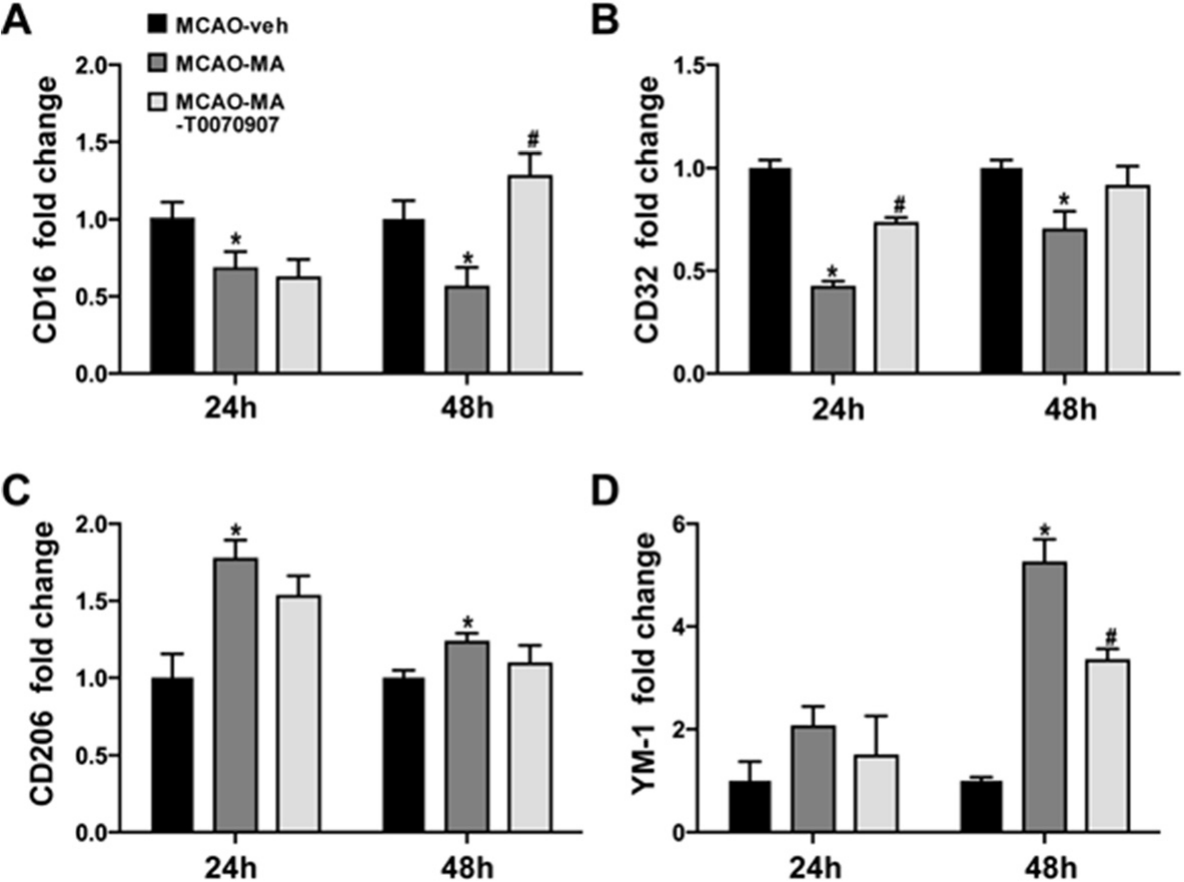
**Effects of PPARγ** **inhibitor T0070907 on microglia/macrophage markers.** Q-PCR for mRNA expression of CD16 **(A)**, CD32 **(B)**, CD206 **(C)**, and YM-1 **(D)** on ipsilateral cortex at 24 and 48 h of MCAO. Quantifications were expressed as the fold change compared to the MCAO-veh group. Values are mean ± SD. ^*^ *P* < 0.05 *vs*. sham; ^#^ *P* < 0.05 *vs*. MCAO-veh & *P* < 0.05 *vs*. MCAO-MA; *n* = 6 in each group.

To explore whether PPARγ inhibitor T0070907 influenced the microglia/macrophage phenotypes, q-PCR was processed. In the MCAO-MA-T0070907 group, M1 marker CD16 and CD32 are significantly higher when compared with the MCAO-MA group at 48 and 24 h, respectively (Figure 8A,B). While M2 marker YM-1 in the MCAO-MA-T0070907 group is decreased significantly comparing with the MCAO-MA group at 48 h (Figure 8D).

## Discussion

In this study, mouse transient cerebral ischemia model is used to demonstrate a novel neuroprotective resveratrol oligomer extraction. We performed a set of experiments to show that MA significantly reduced the infarct size and improved the neural function. In addition, our discoveries also showed that the neuroprotective effects of MA are largely associated with anti-inflammatory effects by activating M2 polarization in a PPARγ-dependent manner.

Even though it is not the first evidence showing that antioxidants may have anti-inflammatory effects, we still performed the experiment in both MCAO model and *in vitro* ways to provide reliable evidence for our hypothesis. In accordance with the *in vivo* results, MA showed strong anti-inflammation effects in LPS-stimulated microglia, which demonstrate a direct function in inhibiting the pro-inflammatory cytokines. On the other hand, we also evaluated the anti-oxidant effects of MA *in vivo* study [20].

In this study, for the MCAO-veh group, M1 genes including CD16, CD32, and CD86 increased soon after stroke and remained high at 72 h. On the other hand, M2 hallmark YM-1 began to decrease 72 h post-ischemia though increasing was found at 48 h. This data is in accordance with that of Hu *et. al*. showing the increase of M1 markers from days 1 to 14 post-ischemia except for CD86, which decreased after day 5,while M2 marks CD206 and YM-1 demonstrated decreasing at earlier time point [21]. Similar trend of CD206 and YM-1 results were also found with immunohistochemistry method in a mice permanent MCAO model [22]. Treatment of MA dampened the upgrading of M1 cytokines but enhanced M2 hallmarks at different time points. However, it might be argued that M1/M2 genes are not expressed only by resident microglia but are also marks of infiltrating peripheral macrophages. The participation of peripheral macrophage cannot be ignored especially when the blood-brain barrier is not intact during brain injury. It is conceded that resident microglia and blood-derived macrophage are indistinguishable functionally and morphologically. However, according to literatures, hematogenous macrophage does not recruit into CNS abundantly until day 4 after stroke [23,24]. Even after day 4, the amount is relatively low compared to resident microglia. Thus, we assumed that the M1/M2 detected in this study is mainly expressed by resident microglia.

PPARγ is a widely expressed nuclear transcriptional factor with well-established protective features. Activation of PPARγ is associated with anti-inflammation in various diseases, including inflammatory bowel disease [25] and ischemia reperfusion-induced kidney injury [26]. Beyond the functions in peripheral systems, PPARγ is also reported as a potential therapeutic target in central nervous system diseases due to its relationship to decreasing pro-inflammatory mediators and improving neurological outcome [27]. PPARγ activation is positively related to ameliorating experimental autoimmune encephalomyelitis (EAE) in mice [28] and reducing amyloid deposition along with improving cognitive function in Alzheimer’s disease models [29]. Liu *et al*. reported suppressed PPARγ expression and activity in rat brain ischemia, and recovered PPARγ was associated with alleviated brain injury [30]. These findings brought up the idea that PPARγ may be a potential therapeutic target after ischemia. In this study, increased PPARγ nuclear translocation and enhanced transcriptional activities after MA treatment indicates potential elevated activation of PPARγ, and the result is further verified by suppression of MA-induced protection through PPARγ inhibitor T0070907.

One of the important roles of PPARγ in the control of inflammation may be related with regulation of M1/M2 phenotype as demonstrated in a post-incisional pain study [31] and Alzheimer’s disease model [29]. Microglia are mostly related to inducing inflammatory cytokines and recruitment of immune cells after ischemic stroke. While microglia is known as a key factor in the rapid, excessive activation of inflammation after CNS injury [32], it remains inconclusive whether microglia has no beneficial effects as it shows some importance in synapse remodeling [33], neurogenesis, and even after stroke [34] until recent research started to focus on the balance of microglia polarization. The evidence of microglia polarization in ischemic stroke models brought up the concept that, compared with general suppression of microglia activation, the inhibition of M1-activated microglia along with encouragement of M2 activation will become a more reasonable treatment target for inflammation-leading diseases [21].

The evidence has shown that activation of PPARγ will increase M2 gene expression in macrophages/microglia, including Arg-1 [35], CD206, and IL-10 [36]. The administration of rosiglitazone increased the expression of M2-specific markers, including Arg1, FIZZ1, and IL-10 [31]. In the current study, MA treatment after MCAO enhanced the activity of PPARγ and further increased its binding activity with M2 genes YM-1 promoter, which will contribute to the enhancement of transcriptional activity of M2 genes. However, PPARγ activation may not be the only way for stimulating M2 gene expression; thus, no difference of binding activity of PPARγ and CD206 was found in MCAO-MA group in this study.

In conclusion, the major discovery of the current study is to demonstrate the protective effects of MA, a novel resveratrol oligomer from *H. hainanensis*, in ischemic stroke by inhibiting inflammation through activation of PPARγ. The effects are related to balancing M1/M2 phenotypes. Our finding suggests that MA is a promising candidate in stroke treatment.
